# Resource use and in-hospital costs after aneurysmal subarachnoid hemorrhage in the Netherlands

**DOI:** 10.1016/j.bas.2025.104400

**Published:** 2025-08-28

**Authors:** F.P. Mulder, J.T.J.M. van Dijck, R.J.G. Vreeburg, C. Engels, W.A. Moojen

**Affiliations:** a Department of Neurosurgery Leiden University Medical Center, Haaglanden Medical Center, Haga Teaching Hospital, University Neurosurgical Center Holland, Leiden and The Hague, the Netherlands; b Department of Interventional Radiology, Haaglanden Medical Center, The Hague, the Netherlands

**Keywords:** Aneurysmal subarachnoid hemorrhage, Cost-driving factors, Healthcare resource consumption, In-hospital costs, Ruptured intracranial aneurysm

## Abstract

**Introduction:**

Aneurysmal Subarachnoid Hemorrhage (aSAH) is a severe condition requiring advanced interventions and intensive in-hospital care. The associated financial burden challenges resource allocation and healthcare sustainability.

**Research question:**

This study aimed to evaluate the in-hospital healthcare consumption and costs for patients hospitalised with aSAH.

**Material and methods:**

A bottom-up approach was used to assess in-hospital healthcare consumption and costs for aSAH patients at a Dutch referral centre (November 2021–April 2024). Costs were calculated by multiplying resource consumption, extracted from electronic health records, by national reference prices, reflecting actual hospital costs rather than reimbursements or billing. A generalized linear model was used to identify key cost determinants.

**Results:**

A total of 147 patients were included. Mean age was 61 (±12) and 72.8 % was female. Median total costs were €31,666 (IQR: €24,167-€50,367) and length of stay was 15 days (IQR: 12–22). Total costs did not differ significantly between clipping (€28,058 (IQR: €24,167-€49,421) and coiling (€30,209 (IQR: €25,482-€50,678). However, procedure costs differed significantly between clipping (€5135 (IQR: €4886-€6000) and coiling (€7159 (IQR: €5583-€8700). The generalized linear model identified World Federation of Neurosurgical Societies grade, length of stay and delayed cerebral ischemia as key determinants of hospital costs.

**Discussion and conclusion:**

In-hospital costs for aSAH patients are substantial, with length of stay, disease severity and complications as key cost drivers. Total in-hospital costs were comparable between clipping and coiling, but procedure-specific costs varied significantly. Understanding cost distribution and cost drivers can support more efficient resource allocation and ultimately improve the cost-effectiveness of aSAH care.

## Highlights

• Median costs of hospital care for aneurysmal subarachnoid hemorrhage were €31,666.
• Length of stay, disease severity and complications are key cost drivers.
• Clipping and coiling have similar total costs despite different procedure costs.

## 1 Introduction

Aneurysmal Subarachnoid Hemorrhage (aSAH) is a leading cause of death and disability worldwide, with an average global incidence of 7.9 per 100,000 persons (Etminan et al., 2019). It is a catastrophic event, carrying a mortality rate of 37 % in Europe. The field of aSAH treatment has seen rapid developments in the last decades with major advancements in neurocritical care, imaging and microsurgical and endovascular techniques (Thilak et al., 2024; Lanzino and Rabinstein, 2024). While these advancements have improved patient outcomes, only 39 %–51 % of all patients achieve functional independence (Ziebart et al., 2024).

The combination of the high incidence, technical advancements in treatment and the post-hospitalization disabilities comes with significant financial implications (Rivero-Arias et al., 2010; Yoon et al., 2019). With rising healthcare expenditures worldwide, this strain threatens the sustainability and affordability of healthcare services (Fiore et al., 2024; Thomson et al., 2009). For example, total direct healthcare costs of aSAH were estimated at £127 million annually in the United Kingdom (2010) and $5.6 billion in lifetime costs in the United States (1990) (Rivero-Arias et al., 2010; Taylor et al., 1996). The direct costs are primarily driven by factors such as advanced medical interventions, postoperative complications and prolonged hospital stays (Bekelis et al., 2016; Mualem et al., 2022).

To maximize the efficiency of allocation of these scarce healthcare resources, it is essential to analyse the healthcare resource consumption and the associated costs (Drummond et al., 2015; World Health Organization, 2010). However, the limited available studies mainly focus on hospital charges, reimbursements or billing rather than using a bottom-up approach to comprehensively assess the actual resource utilization and true costs of aSAH. Moreover, economic evaluation studies often widely vary in methodological quality with a lack of standardisation and transparency in reporting (Xu et al., 2021; Caulley et al., 2020; Emerson et al., 2019; Husereau et al., 2022).

This highlights the need for high quality economic evaluations of aSAH based on actual costs, rather than billing, to guide policymakers and healthcare professionals in delivering optimal and economically sustainable care (Roberts et al., 2019; Chalkidou et al., 2016; Glassman et al., 2012). Therefore, the objective of this study is to analyse the in-hospital resource use and actual hospital costs in patients with aneurysmal subarachnoid hemorrhage and to identify the key cost drivers, providing a foundation for future multicentre cost-effectiveness research.

## 2 Methods

This study adhered to the recommendations outlined in the Consolidated Health Economic Evaluation Reporting Standards (CHEERS) statement (Husereau et al., 2022).

### 2.1 Study design and population

This study included aSAH patients from one participating centre in the multicentre, prospective, observational Study on Prognosis of Acutely Ruptured Intracranial Aneurysms (SPARTA) (Hamming et al., 2024). Participants were enrolled between November 2021 and April 2024 in Haaglanden Medical Center, a regional referral hospital for aSAH patients. The main objective of SPARTA is to determine the effectiveness of all treatment types (coiling or clipping) compared to best supportive care on mortality and long-term outcomes in aSAH patients. The present study is the first economic analysis within the SPARTA cohort, focussing on quantifying in-hospital healthcare consumption and actual costs to support future multicentre cost-effectiveness evaluations. The study was approved by the Medical Ethics Committee Leiden The Hague Delft (METC LDD) and patients or proxies provided informed consent for study participation.

Patients were included in the SPARTA study according to the following inclusion criteria: (1) confirmed diagnosis of subarachnoid hemorrhage on CT-scan or lumbar puncture, (2) intracranial aneurysm proven within 6 months to be the cause of the subarachnoid hemorrhage, (3) age 18 years or over at presentation, (4) written informed consent. Patients were excluded if the subarachnoid hemorrhage was perimesencephalic or post-traumatic, due to intracerebral arteriovenous malformations or dural arteriovenous fistulae or if they had insufficient Dutch or English proficiency.

### 2.2 Data collection

Healthcare resource consumption was analysed using a bottom-up approach (Brouwer et al., 2001). For all individual patients, the consumed units were retrieved from the electronic health records using a predefined, anonymized data extraction form. Healthcare use was categorized by admission, surgical and endovascular procedures, imaging, ambulance transportation, consultations and laboratory tests. Demographic and clinical data were obtained from the SPARTA database. The data included patient age, sex, smoking history, World Federation of Neurosurgical Societies (WFNS) grade at admission, Hunt and Hess (H&H) grade at admission, Modified Fisher Score, size of the aneurysm, location of the aneurysm, type of aneurysm, treatment modality, relevant medical history and in-hospital complications (Hamming et al., 2024).

### 2.3 In-hospital costs

In-hospital costs were calculated from a healthcare perspective according to the guidelines from the Dutch National Health Care Institute (Hakkaart-van Roijen et al., 2024). This entailed a bottom-up micro-costing approach in which consumed healthcare units were multiplied by their respective reference prices. These calculations reflect actual hospital costs based on resource use, not reimbursement tariffs or hospital billing. The reference prices provided in the guideline were primarily used for the calculations (Supplementary Table 1). If the corresponding reference prices were not provided, the average national retail price was retrieved from the Dutch Healthcare Authority. (Nederlandse Zorgautoriteit (NZa))

Applying a standard reference or retail price would not adequately reflect the true costs of the surgical and endovascular procedures due to the variability in costs per procedure. Therefore, all surgical and endovascular procedures were individually assessed to quantify procedure duration and the use of personnel, devices, instrumentation and consumables. Used resources were multiplied by their respective unit costs, obtained from the hospital's procurement records, thereby reflecting the actual hospital costs for these resources. Procedure duration was multiplied by the cost per minute of the operating room, differentiating between a standard, hybrid or neuro-angio operating room (Patel et al., 2022).

All costs were calculated in Euros and converted to 2024 values using the Consumer Price Index provided by the Central Bureau of Statistics in the Netherlands (average exchange rate in 2024: EUR 1 = USD 1.08). (Centraal Bureau voor de Statistiek) (European Central Bank)

### 2.4 Statistical analysis

Baseline patient characteristics are displayed using descriptive statistics including means with standard deviations (SD), medians with interquartile ranges (IQR) and percentages. Subgroup analyses were performed based on various clinical and demographic factors, including age, sex, relevant medical history, aSAH severity, clinical characteristics and treatment modality. Mann-Whitney U tests and Kruskal-Wallis tests were performed to test for differences in total in-hospital costs between the subgroups.

A Generalized Linear Model (GLM) with a Tweedie distribution (power = 1.7) and a log link function was used to identify key predictors of total in-hospital costs. The Tweedie distribution was selected due to its suitability to handle non-negative, right-skewed cost data (Kurz, 2017). A sensitivity analysis compared the Tweedie model with a GLM using a Gamma distribution and also tested alternative Tweedie power parameters. Model fit was evaluated using deviance, Pearson residuals, Akaike's Information Criterion (AIC), and Bayesian Information Criterion (BIC), with the Tweedie model (power = 1.7) providing the best fit. Variables were selected based on clinical relevance and prior literature on cost drivers in aSAH. These included age, sex, relevant medical history, aneurysm characteristics, WFNS scale, complications, treatment modality and hospital length of stay (LOS). To assess for multicollinearity, variance inflation factors (VIF) were calculated. No variables exceeded a VIF of 5, indicating acceptable levels of collinearity. No imputation was necessary, as there were no missing data in variables used for the analysis. Results were reported as regression estimates (β), standard errors (SE), 95 % confidence intervals (CI) and p-values. Adjusted mean estimates of the total in-hospital costs (estimated marginal means, EMM) with 95 % CIs were calculated for the categorical variables. A p-value of <0.05 was considered statistically significant. Statistical analyses were performed using IBM SPSS Statistics version 28.0.

## 3 Results

A total of 147 patients were included. The mean age was 61 (±12) years and 107 (72.8 %) patients were female (Table 1). Among these patients, 36.1 % were current smokers and 10.2 % were former smokers. The most common comorbidity was hypertension (34.0 %) and 6.1 % of the patients had a history of aSAH. The majority of patients (76.2 %) were admitted with WFNS grade I-III. Similarly, 74.1 % of the patients presented with Hunt and Hess grade I-III. Due to delayed imaging, 5 patients were classified with a modified Fisher grade of 0, while the remaining patients were distributed across grades 1 to 4 (26.5 %, 13.6 %, 21.1 % and 35.4 %, respectively).

**Table 1:** Patient characteristics.

Characteristic	All (*N* = 147)	
**Female**	107 (72.8 %)	
**Age**	61.00 (±12.49)	
**Clinical Severity**	**Hunt & Hess**	**WFNS**
I	46 (31.3 %)	77 (52.4 %)
II	47 (32.0 %)	28 (19.0 %)
III	16 (10.9 %)	7 (4.8 %)
IV	8 (5.4 %)	9 (6.1 %)
V	30 (20.4 %)	26 (17.7 %)
**Modified Fisher**		
0	5 (3.4%)	
I	39 (26.5%)	
II	20 (13.6%)	
III	31 (21.1%)	
IV	52 (35.4%)	
**Parenchymal Hemorrhage**	20 (13.6 %)	
**Subdural Hemorrhage**	4 (2.7 %)	
**Location Aneurysm**		
**Anterior Circulation**	**121 (82.3 %)**	
**Posterior Circulation**	**26 (17.7 %)**	
ACOM	51 (34.7 %)	
PCOM	28 (19.0 %)	
MCA	20 (13.6 %)	
ICA	10 (6.8 %)	
BA	13 (8.8 %)	
Other	25 (17.0 %)	
**Size (mm)**	6.49 (±4.66)	
**Type**		
Saccular	122 (83.0 %)	
Dissection	17 (11.6 %)	
Other	8 (5.4 %)	
**Treatment**		
Clipping	27 (18.4 %)	
Endovascular:	101 (68.7 %)	
Coiling	70 (47.6 %)	
Stent-assisted Coiling	15 (10.2 %)	
Flow diversion	16 (10.9 %)	
Both	5 (3.4 %)	
No treatment	14 (9.5 %)	
**Medical History**		
Hypertension	50 (34.0 %)	
aSAH	9 (6.1 %)	
MI	9 (6.1 %)	
**Smoker**		
No	71 (48.3 %)	
Yes	53 (36.1 %)	
Former	15 (10.2 %)	
Unknown	8 (5.4 %)	
**In-hospital Mortality**	32 (21.8 %)	
**Complications**		
Hydrocephalus	62 (42.2 %)	
DCI	32 (21.8 %)	
Rebleed	19 (12.9 %)	
Meningitis	10 (6.8 %)	
Pneumonia	9 (6.1 %)	

Values are presented as absolute numbers (percentages) or mean (±standard deviation). Patient characteristics by treatment modality are available in Supplementary Table 2.

Abbreviations: **WFNS** World Federation of Neurosurgical Societies**, ACOM** Anterior Communicating Artery**, PCOM** Posterior Communicating Artery**, MCA** Middle Cerebral Artery**, ICA** Internal Carotid Artery**, BA** Basilar Artery**, aSAH** Aneurysmal Subarachnoid Hemorrhage**, MI** Myocardial Infarction, **DCI** Delayed Cerebral Ischemia.

The mean size of aneurysms was 6.49 (±4.66) mm with the most common locations being the anterior and posterior communicating arteries (34.7 % and 19.0 %, respectively). These aneurysms were predominantly saccular (83.0 %), while fewer were dissecting (11.6 %) or other rarer types (Table 1). The most common complications were hydrocephalus (42.2 %), delayed cerebral ischemia (21.8 %), rebleed (12.9 %), meningitis (6.8 %) and pneumonia (6.1 %). Moreover, in hospital mortality was 21.8 % (*N* = 32).

### 3.1 In-hospital costs and length of stay

The mean total in-hospital costs for all patients were €41,888 (±€29,600) with a median of €31,666 (IQR: €24,167-€50,367). The largest contributors were costs of admission and surgical and endovascular procedures (55 % and 31 %, respectively). Smaller contributors to the total costs included radiology (5 %), laboratory works (3 %), paramedical care (2 %), transportation (2 %), consultation (1 %) and other miscellaneous expenses (1 %)(Table 2). The mean length of stay was 19 (±15) days with a median of 15 days (12–22). On average, patients spent 4 days (±5 days) on the ICU, 2 days (±3 days) on the Neurocare department and 13 days (±13 days) on the ward (Table 2).

**Table 2:** In-hospital costs and Length of Stay.

Category	Mean (±SD)	Median (IQR)
**Total In-Hospital Costs**	41,888 (±29,600)	31,666 (24,167–50,367)
**Breakdown of Costs:**		
Admission	23,018 (±18,552)	17,950 (11,334–30,185)
Procedures	12,909 (±10,794)	9730 (5503–16,736)
Imaging	1982 (±1701)	1368 (1011–2384)
Laboratory	1258 (±1397)	786 (447–1530)
Transportation	739 (±832)	705 (314–1019)
Paramedical Care	775 (±774)	571 (300–952)
Consultation	679 (±621)	541 (216–916)
Miscellaneous	527 (±504)	515 (129–773)

**Total LOS**	19 (±15)	15 (12–22)
LOS ICU	4 (±5)	3 (2–5)
LOS Neurocare	2 (±3)	0 (0–2)
LOS Ward	13 (±13)	11 (5–15)

Abbreviations: **SD** Standard Deviation, **IQR** Interquartile Range, **LOS** Length of Stay, **ICU** Intensive Care Unit.

### 3.2 Treatment modality

The majority of aneurysms (68.7 %) was secured by endovascular procedures (Coiling (47.6 %), stent-assisted coiling (10.2 %) and flow diversion (10.9 %)), while in 18.4 % microsurgical clipping was performed. Furthermore, 5 patients (3.4 %) underwent both clipping and coiling procedures and 14 patients (9.5 %) did not undergo a surgical or endovascular procedure (Table 1). Cerebrospinal fluid diversion therapy was performed in 38.1 % of patients (*N =* 56)(Supplementary Table 2).

**Fig. 1:**
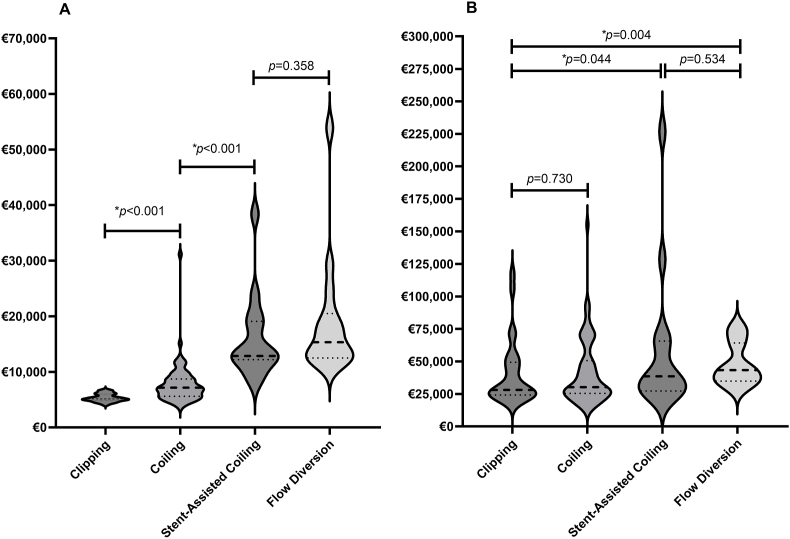
Violin plots showing the distribution of procedural costs (A) and total in-hospital costs (B) across aneurysm treatment modalities. Each plot displays the median (solid line) and interquartile range (dotted lines). P-values in (A) were calculated using the Kruskal-Wallis test; p-values in (B) are based on the Generalized Linear Model (Table 5). Asterisks (∗) indicate statistical significance at p < 0.05.

Median procedure costs differed significantly between the treatment modalities, with clipping being the least expensive (€5135, IQR: €4886-€6000), followed by coiling (€7159, IQR: €5583-€8700), stent-assisted coiling (€12,863, IQR: €12,182-€19,073) and flow diversion (€15,325, IQR: €12,479-€20,497)(Table 3, Fig. 1). However, total in-hospital costs between clipping and coiling were not significantly different (€28,058, IQR: €24,167-€49,421 vs. €30,209, IQR: €25,482-€50,678, respectively). In contrast, stent-assisted coiling and flow diversion were associated with higher total costs (€38,585, IQR: €27,292-€65,646 and €43,328, IQR: €34,904-€64,221, respectively), and were significantly more expensive than clipping according to the generalized linear model (Fig. 1).

**Table 3:** Comparative cost and length of stay by treatment modality.

	Clipping (*N* = 27)	Coiling (*N* = 70)	Stent-assisted Coiling (*N* = 15)	Flow diversion (*N* = 16)	*p*-value
Procedure Cost	5325 (±715)	7730 (±3636)	15,986 (±7587)	18,872 (±10,638)	<0.001∗
	5135 (4886–6000)	7159 (5583–8700)	12,863 (12,182–19,073)	15,325 (12,479–20,497)	
					
Total In-Hospital Costs	40,483 (±25,293)	41,165 (±24,916)	57,893 (±54,236)	48,026 (±16,595)	0.085
	28,058 (24,167–49,421)	30,209 (25,482–50,678)	38,585 (27,292–65,646)	43,328 (34,904–64,221)	
					
Total LOS	22 (±16)	20 (±14)	23 (±21)	16 (±6)	0.732
	17 (14–27)	16 (13–25)	15 (12–20)	16 (13–17)	
					
LOS ICU	5 (±4)	4 (±3)	6 (±10)	4 (±4)	0.331
	4 (2–6)	3 (1–5)	2 (0–4)	3 (0–6)	

Values are reported as mean (±SD) and median (IQR 25–75). Abbreviations: **WFNS** World Federation of Neurosurgical Societies, **aSAH** Aneurysmal Subarachnoid Hemorrhage. *p*-values were calculated using the Kruskal-Wallis test to compare the procedure costs, total in-hospital costs and LOS. Asterisks (∗) indicate statistical significance at p < 0.05. Costs are reported in 2024 Euros and rounded to the nearest Euro. Patients who did not receive treatment to secure the aneurysm (*N* = 14) or those who received both clipping and coiling (*N* = 5) were not included in this analysis.

### 3.3 Cost determinants

Complications such as delayed cerebral ischemia (median: €65,583 vs. €27,833), hydrocephalus (€45,180 vs. €27,292), meningitis (€72,276 vs. €30,245) and pneumonia (€60,321 vs. €30,192) were significant cost drivers (Table 4). Moreover, a history of hypertension (€39,560 vs. €29,264) was associated with higher median in-hospital costs and was identified as a significant cost driver in the GLM (Table 5, Supplementary Table 3). Length of stay was one of the strongest predictors of higher in-hospital costs, with each additional day increasing total costs by 2.8 % (β = 0.028, 95 % CI: 0.024–0.033, *p* < 0.001)(Table 5). Age also influenced total costs, with the highest costs observed in patients aged 40–59 years. WFNS grade had a significant effect on in-hospital costs in the GLM. Grade III had the highest unadjusted median costs (€71,699; IQR: €49,421–€82,432), while grade V showed the highest adjusted costs in the GLM. Deceased patients had significantly shorter hospital stays (4 vs. 16 days) and lower median costs (€24,474 vs. €34,450), however, the overall effect of in-hospital mortality on total costs was not significant in the GLM. In addition, sex, year of admission, aneurysm location, type and size did not have a significant impact on the total in-hospital costs (Table 5, Supplementary Table 3).

**Table 4:** Subgroup analysis of in-hospital costs and length of stay.

Subgroup	*N*	Total LOS	Total in-hospital Cost	*p*-value
**Age**				
18–39	5 (3.4 %)	13 (13–15)	25,244; 27,044 (19,800–29,789)	0.026∗
40–59	61 (41.5 %)	15 (12–22)	44,915; 34,450 (26,700–54,445)	
60–79	70 (47.6 %)	16 (12–25)	43,258; 33,701 (23,624–61,138)	
≥80	11 (7.5 %)	8 (3–23)	23,944; 17,416 (8247–43,380)	
**Sex**				
Male	40 (27.2 %)	16 (12–23)	47,552; 34,865 (24,414–64,402)	0.454
Female	107 (72.8 %)	15 (10–22)	39,770; 30,245 (24,167–49,984)	
**WFNS**				
I	77 (52.4 %)	15 (13–19)	34,563; 28,515 (24,131–42,134)	0.041∗
II	28 (19.0 %)	17 (13–23)	45,280; 35,676 (26,645–46,224)	
III	7 (4.8 %)	29 (15–39)	65,480; 71,699 (49,421–82,432)	
IV	9 (6.1 %)	34 (3–46)	46,033; 49,984 (13,840–70,557)	
V	26 (17.7 %)	12 (3–25)	52,139; 44,344 (19,635–72,354)	
**Modified Fisher**				
0	5 (3.4 %)	9 (6–19)	32,988; 26,445 (16,855–52,393)	0.003∗
1	39 (26.5 %)	14 (12–16)	29,004; 27,255 (21,136–33,281)	
2	20 (13.6 %)	16 (12–20)	36,711; 34,241 (25,509–44,522)	
3	31 (21.1 %)	17 (13–26)	47,970; 43,981 (29,631–68,001)	
4	52 (35.4 %)	16 (4–29)	50,771; 40,809 (26,561–71,010)	
**Location Aneurysm**				
Anterior Circulation	121 (82.3 %)	15 (12–22)	41,125; 30,723 (24,131–50,989)	0.960
Posterior Circulation	26 (17.7 %)	15 (7–23)	45,438; 33,514 (24,265–50,907)	
**In-hospital Mortality**				
No	115 (78.2 %)	16 (14–26)	43,907; 34,450 (26,556–49,984)	0.021∗
Yes	32 (21.8 %)	4 (3–13)	34,630; 24,474 (14,256–61,732)	
**DCI**				
No	115 (78.2 %)	15 (9–18)	33,927; 27,833 (22,271–41,754)	<0.001∗
Yes	32 (21.8 %)	24 (16–46)	70,494; 65,583 (44,921–84,447)	
**Hydrocephalus**				
No	85 (57.8 %)	15 (12–17)	31,719; 27,292 (21,962–38,651)	<0.001∗
Yes	62 (42.2 %)	21 (12–32)	55,829; 45,180 (28,345–72,241)	
**Meningitis**				
No	137 (93.2 %)	15 (11–21)	38,883; 30,245 (23,714–47,962)	<0.001∗
Yes	10 (6.8 %)	34 (18–60)	83,058; 72,276 (40,076–100,582)	
**Pneumonia**				
No	138 (93.9 %)	15 (11–21)	40,441; 30,192 (23,881–47,866)	0.001∗
Yes	9 (6.1 %)	25 (17–38)	64,066; 60,321 (46,876–76,515)	

Values are reported as: absolute numbers (percentages), median (IQR 25–75) or mean; median (IQR 25–75) *p*-values were calculated using the Mann-Whitney *U* test and the Kruskal-Wallis test to compare the total in-hospital costs. Asterisks (∗) indicate statistical significance at p < 0.05. Costs are reported in 2024 Euros and rounded to the nearest Euro.

Abbreviations: **WFNS** World Federation of Neurosurgical Societies, **aSAH** Aneurysmal Subarachnoid Hemorrhage, **DCI** Delayed Cerebral Ischemia.

**Table 5:** Generalized linear model of predictors for total in-hospital costs.

	Estimate (β)	SE	95 % CI		*p*-value	Estimated Marginal Mean Total Cost
			Lower	Upper		
**Intercept**	9.726	0.106	9.519	9.934		

**Total LOS**	0.028	0.003	0.024	0.033	<0.001∗	–
**Size Aneurysm**	0.004	0.004	−0.004	0.011	0.310	–

**Sex**					0.698	
Male	∗∗	–	–	–		54,873 (46,299–65,034)
Female	−0.015	0.039	−0.090	0.061		54,058 (46,410–62,966)
**Age**					<0.001∗	
18–39	∗∗	–	–	–		56,867 (44,676–72,384)
40–59	0.101	0.094	−0.083	0.286		62,930 (53,775–73,644)
60–79	−0.013	0.095	−0.200	0.173		56,110 (48,154–65,382)
≥80	−0.261	0.122	−0.500	−0.021		43,820 (35,983–53,365)
**WFNS**					0.023∗	
I	∗∗	–	–	–		53,551 (45,648–62,823)
II	0.147	0.079	−0.007	0.301		53,101 (44,812–62,923)
III	0.244	0.189	−0.107	0.594		58,771 (46,963–73,548)
IV	−0.098	0.150	−0.393	0.197		48,376 (38,135–61,368)
V	0.213	0.096	0.024	0.402		59,276 (50,348–69,786)
**Treatment Modality**					<0.001∗	
Clipping	∗∗	–	–	–		55,923 (46,837–66,772)
Coiling	−0.016	0.046	−0.107	0.075		55,039 (47,143–64,256)
Stent-assisted Coiling	0.133	0.066	0.005	0.261		63,881 (53,394–76,427)
Flow diversion	0.191	0.066	0.061	0.322		67,723 (57,254–80,106)
Clipping and Coiling	0.270	0.101	0.072	0.467		73,242 (58,469–91,747)
No intervention	−0.737	0.100	−0.932	−0.542		26,763 (20,847–34,358)
**Subdural Hemorrhage**					0.172	
No	∗∗	–	–	–		50,905 (44,521–58,204)
Yes	0.135	0.099	−0.059	0.329		58,272 (46,580–72,899)
**In-hospital Mortality**					0.392	
No	∗∗	–	–	–		45,442 (38,151–54,126)
Yes	0.105	0.122	−0.135	0.344		65,277 (54,693–77,909)
**History of aSAH**					0.112	
No	∗∗	–	–	–		51,493 (44,119–60,100)
Yes	0.112	0.071	−0.026	0.251		57,606 (47,782–69,450)
**History of Hypertension**					0.013∗	
No	∗∗	–	–	–		52,045 (44,379–61,035)
Yes	0.091	0.037	0.019	0.163		56,995 (48,437–67,067)
**DCI**					<0.001∗	
No	∗∗	–	–	–		49,801 (42,561–58,271)
Yes	0.179	0.045	0.091	0.267		59,564 (50,311–70,519)
**Hydrocephalus**					<0.001∗	
No	∗∗	–	–	–		49,812 (42,523–58,349)
Yes	0.179	0.042	0.097	0.260		59,551 (50,437–70,311)
**Pneumonia**					<0.001∗	
No	∗∗	–	–	–		53,384 (45,840–62,168)
Yes	0.623	0.187	0.256	0.990		55,566 (45,189–68,326)

Model fit: Deviance/df (1.000) and the Pearson Chi-Square/df (0.990).

The estimated marginal mean of the total in-hospital cost (95 % CI) was rounded to the nearest Euro (2024 values), with aneurysm size fixed at 6.49 mm and LOS at 19 days.

(∗) Statistical significance was set at p < 0.05.

(∗∗) Reference Category.

Abbreviations: **SE** Standard Error, **CI** Confidence Interval, **LOS** Length of Stay, **aSAH** Aneurysmal Subarachnoid Hemorrhage, **DCI** Delayed Cerebral Ischemia.

## 4 Discussion

This study found that in-hospital costs for aneurysmal subarachnoid hemorrhage are substantial, with admission (55 %) and surgical or endovascular procedures (31 %) accounting for the majority of the total costs. While total in-hospital costs were similar between clipping and coiling, procedural costs varied significantly. Key cost drivers included length of stay, disease severity, complications, and treatment modality.

Our findings aligned with previous European estimates, with reported in-hospital costs ranging from €35,280 to €46,976 (Rivero-Arias et al., 2010; Seule et al., 2020; Calciolari et al., 2013) (2024 values). However, reported costs varied considerably across geographic regions and studies, ranging from €57,057 to €259,564 in the United States (Kainth et al., 2014; Labib et al., 2022; Stepanova et al., 2013) to substantially lower costs in Thailand (€8097), Taiwan (€8472) and Brazil (€10,234) (Duangthongphon et al., 2022; Lee et al., 2013; Safanelli et al., 2019). These differences reflect the variability in prices of healthcare services between countries caused by labour costs, healthcare system design, regulations, reimbursement policies and the overall economic condition (Lorenzoni and Dougherty, 2022; Koechlin et al., 2017). Differences in patient population and study methodology (e.g. charges/reimbursements vs. actual costs, included cost components and economic perspective) further contribute to the variation in reported costs. Additionally, rising costs over time add to this variability, driven by costly advancements in neurocritical care, imaging and endovascular technologies (Thilak et al., 2024; Lanzino and Rabinstein, 2024; Yoon et al., 2019; Modi et al., 2019).

Procedural costs were significantly higher for endovascular procedures, with substantial variation observed within this group. This can be attributed to differences in duration of the procedure, characteristics of the aneurysm, overall complexity of the case and the number of consumables and devices used. In particular, stent-assisted coiling and flow diversion had nearly double the procedural costs compared to standard coiling and were also associated with higher total in-hospital costs. This reflects the complexity of the management of these cases, underscoring the need for future research on the cost-effectiveness of clipping and endovascular procedures.

Despite higher procedural costs for coiling, total in-hospital costs were similar between clipping and standard coiling, likely due to longer total and ICU stays in the neurosurgical group. This is consistent with several studies reporting no significant differences between both treatment modalities (Yoon et al., 2019; Modi et al., 2019; Deutsch et al., 2018; Monsivais et al., 2019), although others report significantly higher costs for clipping (Abecassis et al., 2019; Ridwan et al., 2021) or coiling (Labib et al., 2022; Chang et al., 2016). These discrepancies are a result of differences in aneurysm location (Abecassis et al., 2019), unequal distribution of clinical grades (Ridwan et al., 2021) or the use of hospital billing data instead of actual costs (Chang et al., 2016), highlighting the difficulty of study comparison. Furthermore, while acute costs were comparable, coiling may incur higher long-term costs, due to lower aneurysm occlusion rates and higher rates of rebleeding and retreatment, as suggested by previous systematic reviews (Lindgren et al., 2018; Ahmed et al., 2019).

Length of stay, particularly in the ICU, was among the strongest cost drivers of total in-hospital costs, which is in accordance with the existing literature. Given the high costs of ICU care, several studies have proposed strategies to reduce unnecessary stays to optimize resource allocation (Benevides Santos Paiva et al., 2024; Alaraj et al., 2017). Disease severity was another key determinant of total in-hospital costs. After adjustment in the GLM, WFNS grades III-V were associated with significantly higher costs. Although outcomes have improved over time, higher WFNS grades remain associated with unfavourable prognosis and lower functional recovery after hospitalization, which contributes to a higher total economic burden (Seule et al., 2020; Nguyen et al., 2023).

While deceased patients generally incurred significantly lower costs, the overall effect of mortality on total in-hospital costs was more complex and statistically not significant due to strong influences from LOS, procedural costs and disease severity. Fourteen patients had treatment-limiting decisions made shortly after admission, resulting in minimal care, resource use and subsequently lower costs. In contrast, other deceased patients underwent prolonged intensive care and multiple treatments, resulting in higher costs despite poor outcomes. This is reflected in the higher estimated marginal means for deceased patients in the GLM, highlighting the intensive and resource-demanding care that they required due to the more severe clinical presentation at admission.

Complications such as hydrocephalus, pneumonia and delayed cerebral ischemia were key cost drivers, primarily due to prolonged (ICU) stay and additional medical interventions. For delayed cerebral ischemia, costs were further increased by the need for additional imaging and advanced endovascular therapy, including intra-arterial nimodipine and balloon angioplasty. In addition to direct costs, the consequences of complications, particularly DCI, may lead to long-term disability and increased indirect costs. This warrants the importance of timely detection and effective treatment of complications to improve patient outcome and reduce in-hospital costs.

### 4.1 Limitations

The most important limitation is the underestimation of the total costs caused by not including indirect and intangible costs, such as long-term costs, rehabilitation costs and costs related to loss of productivity. The costs of productivity loss are particularly high for aSAH patients due to the relatively young age of onset, subsequently resulting in a significant loss in productive working years (Rivero-Arias et al., 2010; Seule et al., 2020). Furthermore, this study did not account for functional outcome and quality-of-life parameters and their relationship to in-hospital costs. Moreover, the small sample size, particularly for less common procedures like flow diversion, may limit the statistical power to draw definitive conclusions. In addition, the single centre design of this study could have impacted the generalizability and applicability of the results outside the Dutch healthcare context due to the known differences in healthcare systems, reimbursement models, treatment variation between centres and differences in procurement and industry partners (Dijkland et al., 2020; de Winkel et al., 2021).

To improve generalizability and provide a more comprehensive understanding of the economic burden of aSAH, future research should involve multicentre and long-term economic evaluations including both direct and indirect costs. Additionally, the present data can be used to perform a much-needed cost-effectiveness study comparing treatment modalities within the SPARTA cohort (e.g. open microsurgical versus endovascular treatments). Results could provide valuable information to aid policymakers and healthcare professionals in clinical decision making and resource allocation.

## 5 Conclusion

In-hospital healthcare consumption and costs for aSAH patients are substantial. Total in-hospital costs were similar for clipping and coiling, however, procedure-specific costs varied significantly. Key cost-driving factors are length of stay, disease severity, surgical and endovascular procedures and complications such as delayed cerebral ischemia and hydrocephalus. A better understanding of these cost distributions can support more efficient resource allocation, improve the cost-effectiveness of aSAH care and help to achieve economically sustainable healthcare with high-quality patient management.

## Author contributions

FM and JD contributed to the conceptualization and design of the study. FM collected the data and performed the analysis. All authors contributed to the interpretation of the data and results. FM drafted the manuscript, which was critically reviewed and revised by JD, RV, CE, and WM. All authors approved the final version prior to submission.

## Ethics

The study was conducted in accordance with the principles of the Declaration of Helsinki (last revised in October 2024 at the General Assembly in Helsinki) and the Medical Research Involving Human Subjects Act (WMO). The study was approved by the Medical Ethics Committee Leiden The Hague Delft. Informed consent was obtained from all participants or their legal proxies.

## Funding

The prospective Study on the Prognosis of Acutely Ruptured intracranial Aneurysms (SPARTA) is sponsored by the Sint Jacobus Stichting, a non-profit organisation. The sponsor has no role in the design of the study and the collection, analysis and interpretation of data.

## Conflicts of interest

The authors declare that there is no conflict of interest.

## Abbreviations:

**aSAH** aneurysmal Subarachnoid Hemorrhage, **CI** Confidence Interval, **CT** Computed Tomography, **DCI** Delayed Cerebral Ischemia, **EMM** Estimated Marginal Means, **GLM** Generalized Linear Model, **H&H** Hunt and Hess, **ICU** Intensive Care Unit, **LOS** Length of Stay, **SPARTA** Study on Prognosis of Acutely Ruptured Intracranial Aneurysms, **WFNS** World Federation of Neurosurgical Societies.
