# Supplementary material for: Resource use and in-hospital costs after aneurysmal subarachnoid hemorrhage in the Netherlands
Source: Brain Spine. 2025 Aug 28;5:104400. doi: 10.1016/j.bas.2025.104400 (PMC12451355; doi:10.1016/j.bas.2025.104400)
Supplement: Multimedia component 1 [file mmc1.docx]

| **Supplementary Table 1.** Unit Costs and Reference Prices | | | | |
| --- | --- | --- | --- | --- |
| **Healthcare Consumption Unit** | **Reference Price (2024)** | |  | **Source** |
| **Surgical Procedures:** |  | |  |  |
| Lumbar Punction | 220.06 | | Per Procedure | ^24^ |
| Groin Exploration | 7028.99 | | Per Procedure | ^24^ |
| Tracheostomy | 1835.05 | | Per Procedure | ^24^ |
| **Imaging:** |  | |  |  |
| MRI Cerebrum | 272.57 | | Per Unit | ^23^ |
| MRI Cervical Spine | 272.57 | | Per Unit | ^23^ |
| CT Cerebrum | 164.19 | | Per Unit | ^23^ |
| CT Cervical Spine | | 201.75 | Per Unit | ^23^ |
| CT Thorax | | 210.33 | Per Unit | ^23^ |
| CT Abdomen | | 229.65 | Per Unit | ^23^ |
| X-ray Skull | | 88.18 | Per Unit | ^23^ |
| X-ray Thorax | | 88.18 | Per Unit | ^23^ |
| X-ray Abdomen | | 88.18 | Per Unit | ^23^ |
| Echography Abdomen | | 126.63 | Per Unit | ^23^ |
| Echography Upper Extremities | | 110.53 | Per Unit | ^23^ |
| Echography Lower Extremities | | 104.27 | Per Unit | ^23^ |
| Echocardiography | | 58.57 | Per Unit | ^23^ |
| **Transportation:** | |  |  |  |
| Ambulance Emergency | | 705.05 | Per Unit | ^23^ |
| Ambulance Non-Emergency | | 314.43 | Per Unit | ^23^ |
| Mobile Medical Team | | 4760.10 | Per Unit | * |
| **Admission:** | |  |  |  |
| Emergency Room Admission | | 276.87 | Per Visit | ^23^ |
| ICU Admission | | 2926.42 | Per Day | ^23^ |
| Ward Admission | | 691.09 | Per Day | ^23^ |
| **Consultation:** | |  | | |
| Neurology | | 155.60 | ** | ^24^ |
| Psychiatry | | 257.55 | ** | ^24^ |
| Surgery (General) | | 123.41 | ** | ^24^ |
| Cardiology | | 123.41 | ** | ^24^ |
| Internal Medicine | | 134.14 | ** | ^24^ |
| Anaesthesiology | | 171.70 | ** | ^24^ |
| Revalidation | | 372.38 | ** | ^24^ |
| Ophthalmology | | 144.87 | ** | ^24^ |
| Urologist | | 139.51 | ** | ^24^ |
| Plastic Surgery | | 155.60 | ** | ^24^ |
| Gynaecology | | 155.60 | ** | ^24^ |
| ENT | | 107.31 | ** | ^24^ |
| Dermatology | | 134.14 | ** | ^24^ |
| **Paramedical Care:** | |  |  |  |
| Physical therapy | | 41.73 | Per Visit | ^23^ |
| Speech therapy | | 43.92 | Per Visit | ^23^ |
| Dietitian | | 26.51 | Per Visit | ^23^ |
| Ergotherapy | | 26.10 | Per Visit | ^23^ |
| Social Worker | | 136.29 | Per Visit | ^24^ |
| **Lab Works:** | |  |  |  |
| Sodium | | 1.81 | Per Unit | ^54^ |
| Potassium | | 1.79 | Per Unit | ^54^ |
| Chloride | | 1.45 | Per Unit | ^54^ |
| Calcium | | 1.80 | Per Unit | ^54^ |
| Magnesium | | 2.94 | Per Unit | ^54^ |
| Phosphate | | 1.88 | Per Unit | ^54^ |
| Urea | | 1.68 | Per Unit | ^54^ |
| Creatinine/eGFR | | 8.19 | Per Unit | ^54^ |
| Glucose | | 1.80 | Per Unit | ^54^ |
| Bilirubin | | 1.64 | Per Unit | ^54^ |
| Albumin | | 1.66 | Per Unit | ^54^ |
| Protein | | 1.79 | Per Unit | ^54^ |
| Alkaline Phosphatase | | 1.99 | Per Unit | ^54^ |
| Gamma-Glutamyltransferase | | 1.96 | Per Unit | ^54^ |
| Aspartate Aminotransferase | | 1.99 | Per Unit | ^54^ |
| Alanine Aminotransferase | | 1.87 | Per Unit | ^54^ |
| Lactate Dehydrogenase | | 13.70 | Per Unit | ^54^ |
| Creatine Kinase | | 1.99 | Per Unit | ^54^ |
| Troponin T | | 9.34 | Per Unit | ^54^ |
| Glucose | | 1.80 | Per Unit | ^54^ |
| HbA1c | | 4.85 | Per Unit | ^54^ |
| C-Reactive Protein | | 4.70 | Per Unit | ^54^ |
| Erythrocyte Sedimentation Rate | | 2.11 | Per Unit | ^54^ |
| Thyroid-stimulating hormone | | 4.44 | Per Unit | ^54^ |
| T3 | | 7.27 | Per Unit | ^54^ |
| T4 | | 4.28 | Per Unit | ^54^ |
| Cortisol | | 7.14 | Per Unit | ^54^ |
| Vitamin B12 | | 7.06 | Per Unit | ^54^ |
| Hemoglobin / Hematocrit / Cell Indices | | 1.90 | Per Unit | ^54^ |
| Leukocytes | | 1.97 | Per Unit | ^54^ |
| Thrombocytes | | 1.96 | Per Unit | ^54^ |
| Blood Typing | | 5.49 | Per Unit | ^54^ |
| Methemoglobin | | 3.31 | Per Unit | ^54^ |
| D-Dimer | | 11.12 | Per Unit | ^54^ |
| Prothrombin Time Test | | 4.63 | Per Unit | ^54^ |
| Activated Partial Thromboplastin Time | | 4.19 | Per Unit | ^54^ |
| Fibrinogen | | 5.32 | Per Unit | ^54^ |
| Blood Gas | | 5.74 | Per Unit | ^54^ |
| pH | | 3.18 | Per Unit | ^54^ |
| Saturation | | 4.82 | Per Unit | ^54^ |
| Carbon Monoxide | | 6.46 | Per Unit | ^54^ |
| Iron | | 2.49 | Per Unit | ^54^ |
| Transferrin | | 4.67 | Per Unit | ^54^ |
| Ferritin | | 6.86 | Per Unit | ^54^ |
| Blood Culture | | 33.99 | Per Unit | ^54^ |
| Urine Sediment | | 2.79 | Per Unit | ^54^ |
| Gram Stain | | 7.17 | Per Unit | ^54^ |
| Cell Culture | | 15.51 | Per Unit | ^54^ |
| Determination Culture | | 10.36 | Per Unit | ^54^ |
| Antimicrobial Assay | | 10.68 | Per Unit | ^54^ |
| Resistance Characterization (qualitative) | | 8.37 | Per Unit | ^54^ |
| Resistance Characterization (quantitative) | | 11.65 | Per Unit | ^54^ |
| Urine Screening | | 2.74 | Per Unit | ^54^ |
| Fecal Calcium | | 5.07 | Per Unit | ^54^ |
| Hybridization | | 26.13 | Per Unit | ^54^ |
| RNA Amplification (quantitative) | | 184.20 | Per Unit | ^54^ |
| RNA Amplification (qualitative) | | 102.27 | Per Unit | ^54^ |
| DNA Amplification | | 75.56 | Per Unit | ^54^ |
| Osmolarity | | 4.46 | Per Unit | ^54^ |
| Microscopy – Punctates | | 8.76 | Per Unit | ^54^ |
| Order tariff | | 4.53 | Per Unit | ^54^ |
| **Legend:**  **Supplementary Table 1.** This table presents a detailed overview of the unit costs and reference prices used for the cost calculations. Healthcare services are categorized by surgical procedures, diagnostic imaging, patient transportation, admissions, consultations, paramedical care, and laboratory tests. Costs are reported in 2024 Euros.  Abbreviations: **MRI** Magnetic Resonance, Imaging, **CT** Computed Tomography,  **ICU** Intensive Care Unit, **ENT** Ear, Nose, and Throat, **eGFR** Estimated Glomerular Filtration Rate, **HbA1c** Hemoglobin A1c, **CRP** C-Reactive Protein, **ESR** Erythrocyte Sedimentation Rate, **T3** Triiodothyronine, **T4** Thyroxine, **AST** Aspartate Aminotransferase, **ALT** Alanine Aminotransferase, **LDH** Lactate Dehydrogenase, **CK** Creatine Kinase, **TSH** Thyroid-Stimulating Hormone, **PT** Prothrombin Time, **aPTT** Activated Partial Thromboplastin Time, **CO** Carbon Monoxide, **RNA** Ribonucleic Acid, **DNA** Deoxyribonucleic Acid  ***** The unit cost was derived from national expenditure data and adjusted to 2024 values. In 2011, the total national cost associated with the intervention was €21.3 million, based on 6220 reported uses, resulting in a unit cost of €3424.^52,53^ To account for inflation, the cost was adjusted using the Dutch Consumer Price Index. This yielded an updated 2024 unit cost of approximately €4760.10 per unit.  ** Consultation with other medical specialists, regardless of the underlying diagnosis or indication, was recorded as a single visit. | | | | |

**References**

23. Hakkaart-van Roijen L, Peeters S, Kanters T, et al. *Kostenhandleiding voor economische evaluaties in de gezondheidszorg: Methodologie en Referentieprijzen.* Herziene versie 2024 ed. Zorginstituut Nederland; 2024.

24. NZa zorgproductapplicatie. Nza.nl. Accessed October 5, 2024. https://zorgproducten.nza.nl/ZoekZorgproduct.aspx

52. RechtNet BV. Login pagina - Recht.nl. Recht.nl. Accessed October 5, 2024. <https://www.recht.nl/rechtspraak/uitspraak/?ecli=ECLI:NL:CBB:2015:428>

53. Ambulancezorg Nederland. Ambulances in Zicht 2015: Kengetallen en trends van de ambulancezorg in Nederland. Ambulancezorg Nederland; 2015. Accessed October 5, 2024. <https://www.ambulancezorg.nl/static/upload/raw/1aa59bb7-a8e0-48e5-897e-06f9703a7e3d/ambulances-in-zicht-2015.pdf>

54. Medisch-specialistische zorg. Nza.nl. November 23, 2017. Accessed October 5, 2024. https://www.nza.nl/zorgsectoren/medisch-specialistische-zorg
