# Supplementary material for: Resource use and in-hospital costs after aneurysmal subarachnoid hemorrhage in the Netherlands
Source: Brain Spine. 2025 Aug 28;5:104400. doi: 10.1016/j.bas.2025.104400 (PMC12451355; doi:10.1016/j.bas.2025.104400)
Supplement: Multimedia component 2 [file mmc2.docx]

| **Supplementary Table 2. Patient Characteristics by Treatment Modality** | | | | | | |
| --- | --- | --- | --- | --- | --- | --- |
| **Characteristic** | **All (*N*=147)** | **Clipping (*N*=27)** | **Coiling (*N*=70)** | **Stent-assisted Coiling (*N*=15)** | **Flow diversion**  **(*N*=16)** | ***p*-value** |
| **Female** | 107 (72.8%) | 19 (70.4%) | 54 (77.1%) | 9 (60.0%) | 11 (68.8%) | 0.549 |
| **Age** | **61.00 (±12.49)** | **60.30 (±11.84)** | **60.43 (±12.07)** | **58.20 (±11.61)** | **57.94 (±11.76)** | 0.825 |
| 18-39 | 5 (3.4%) | 1 (3.7%) | 3 (4.3%) | - | 1 (6.3%) |  |
| 40-59 | 61 (41.5%) | 12 (44.4%) | 28 (40.0%) | 9 (60.0%) | 7 (43.8%) |  |
| 60-79 | 70 (47.6%) | 13 (48.1%) | 35 (50.0%) | 5 (33.3%) | 8 (50.0%) |  |
| ≥80 | 11 (7.5%) | 1 (3.7%) | 4 (5.7%) | 1 (6.7%) | - |  |
| **WFNS** |  |  |  |  |  | 0.599 |
| I | 77 (52.4%) | 16 (59.3%) | 39 (55.7%) | 11 (73.3%) | 8 (50.0%) |  |
| II | 28 (19.0%) | 4 (14.8%) | 14 (20.0%) | 1 (6.7%) | 6 (37.5%) |  |
| III | 7 (4.8%) | 2 (7.4%) | 2 (2.9%) | 1 (6.7%) | - |  |
| IV | 9 (6.1%) | 1 (3.7%) | 6 (8.6%) | - | - |  |
| V | 26 (17.7%) | 4 (14.8%) | 9 (12.9%) | 2 (13.3%) | 2 (12.5%) |  |
| **Hunt & Hess** |  |  |  |  |  | 0.152 |
| I | 46 (31.3%) | 11 (40.7%) | 28 (40.0%) | 2 (13.3%) | 3 (18.8%) |  |
| II | 47 (32.0%) | 7 (25.9%) | 20 (28.6%) | 8 (53.3%) | 10 (62.5%) |  |
| III | 16 (10.9%) | 2 (7.4%) | 5 (7.1%) | 3 (20.0%) | 2 (12.5%) |  |
| IV | 8 (5.4%) | 3 (11.1%) | 5 (7.1%) | - | - |  |
| V | 30 (20.4%) | 4 (14.8%) | 12 (17.1%) | 2 (13.3%) | 1 (6.3%) |  |
| **Modified Fisher** |  |  |  |  |  | 0.611 |
| 0 | 5 (3.4%) | 1 (3.7%) | 4 (5.7%) | - | - |  |
| 1 | 39 (26.5%) | 9 (33.3%) | 18 (25.7%) | 6 (40.0%) | 5 (31.3%) |  |
| 2 | 20 (13.6%) | 2 (7.4%) | 11 (15.7%) | 2 (13.3%) | 3 (18.8%) |  |
| 3 | 31 (21.1%) | 7 (25.9%) | 14 (20.0%) | 1 (6.7%) | 6 (37.5%) |  |
| 4 | 52 (35.4%) | 8 (29.6%) | 23 (32.9%) | 6 (40.0%) | 2 (12.5%) |  |
| **Parenchymal Haemorrhage** | 20 (13.6%) | 8 (29.6%) | 5 (7.1%) | 3 (20.0%) | 1 (6.3%) | 0.020* |
| **Subdural Haemorrhage** | 4 (2.7%) | 1 (3.7%) | 2 (2.9%) | 1 (6.7%) | - | 0.757 |
| **Drain** | **56 (38.1%)** | **7 (25.9%)** | **26 (37.1%)** | **5 (33.3%)** | **7 (43.8%)** | 0.644 |
| EVD | 31 (21.1%) | 3 (11.1%) | 15 (21.4%) | 1 (6.7%) | 3 (18.8%) |  |
| ELD | 6 (4.1%) | - | 1 (1.4%) | 2 (13.3%) | 3 (18.8%) |  |
| VPD | 4 (2.7%) | 1 (3.7%) | 3 (4.3%) | - | - |  |
| Combination | 15 (10.2%) | 3 (11.1%) | 7 (10.0%) | 2 (13.3%) | 1 (6.3%) |  |
| **Location Aneurysm** |  |  |  |  |  | <.001* |
| **Anterior Circulation** | **121 (82.3%)** | **26 (96.3%)** | **65 (92.9%)** | **8 (53.3%)** | **9 (56.3%)** |  |
| **Posterior Circulation** | **26 (17.7%)** | **1 (3.7%)** | **5 (7.1%)** | **7 (46.7%)** | **7 (43.8%)** |  |
| ACOM | 51 (34.7%) | 4 (14.8%) | 31 (44.3%) | 7 (46.7%) | 1 (6.3%) |  |
| PCOM | 28 (19.0%) | 1 (3.7%) | 21 (30.0%) | - | 3 (18.8%) |  |
| ACA | 5 (3.4%) | 1 (3.7%) | 3 (4.3%) | - | - |  |
| PCA | 3 (2.0%) | - | 1 (1.4%) | - | 1 (6.3%) |  |
| MCA | 20 (13.6%) | 19 (70.4%) | - | 1 (6.7%) | - |  |
| ICA | 10 (6.8%) | - | 6 (8.6%) | - | 3 (18.8%) |  |
| BA | 13 (8.8%) | - | 1 (1.4%) | 6 (40.0%) | 2 (12.5%) |  |
| OA | 3 (2.0%) | - | 2 (2.9%) | - | 1 (6.3%) |  |
| SCA | 1 (0.7%) | - | 1 (1.4%) | - | - |  |
| AICA | 1 (0.7%) | - | 1 (1.4%) | - | - |  |
| PICA | 8 (5.4%) | 1 (3.7%) | 1 (1.4%) | 1 (6.7%) | 4 (25.0%) |  |
| PA | 4 (2.7%) | 1 (3.7%) | 2 (2.9%) | - | 1 (6.3%) |  |
| **Size (mm)** | **6.49 (±4.66)** | **6.94 (±5.97)** | **5.79 (±3.22)** | **8.73 (±3.33)** | **4.98 (±4.42)** | 0.008* |
| Small (<5mm) | 59 (40.1%) | 12 (44.4%) | 29 (41.4%) | 2 (13.3%) | 10(62.5%) |  |
| Medium (5mm-10mm) | 64 (43.5%) | 12 (44.4%) | 33 (47.1%) | 6 (40.0%) | 5 (31.3%) |  |
| Large (10mm-25mm) | 24 (16.3%) | 3 (11.1%) | 8 (11.4%) | 7 (46.7%) | 1 (6.3%) |  |
| **Type** |  |  |  |  |  | <0.001* |
| Saccular | 122 (83.0%) | 25 (92.6%) | 65 (92.9%) | 12 (80.0%) | 4 (25.0%) |  |
| Dissection | 17 (11.6%) | - | 4 (5.7%) | 1 (6.7%) | 10 (62.5%) |  |
| Other | 8 (5.4%) | 2 (7.4%) | 1 (1.4%) | 2 (13.3%) | 2 (12.5%) |  |
| **Medical History** |  |  |  |  |  |  |
| aSAH | 9 (6.1%) | 1 (3.7%) | 3 (4.3%) | 1 (6.7%) | 2 (12.5%) | 0.588 |
| Hypertension | 50 (34.0%) | 5 (18.5%) | 28 (40.0%) | 6 (40.0%) | 4 (25.0%) | 0.184 |
| MI | 9 (6.1%) | - | 6 (8.6%) | - | 2 (12.5%) | 0.211 |
| **Smoker** |  |  |  |  |  | 0.407 |
| No | 71 (48.3%) | 12 (44.4%) | 33 (47.1%) | 9 (60.0%) | 8 (50.0%) |  |
| Yes | 53 (36.1%) | 11 (40.7%) | 29 (41.4%) | 4 (26.7%) | 3 (18.8%) |  |
| Former | 15 (10.2%) | 4 (14.8%) | 6 (8..6%) | 1 (6.7%) | 3 (18.8%) |  |
| Unknown | 8 (5.4%) | - | 2 (2.9%) | 1 (6.7%) | 2 (12.5%) |  |
| **In-hospital Mortality** | 32 (21.8%) | 2 (7.4%) | 12 (17.1%) | 1 (6.7%) | 3 (18.8%) | 0.474 |
| **Complications** |  |  |  |  |  |  |
| Hydrocephalus | 62 (42.2%) | 8 (29.6%) | 31 (44.3%) | 6 (40.0%) | 6 (37.5%) | 0.617 |
| DCI | 26 (17.7%) | 4 (14.8%) | 15 (21.4%) | 3 (20.0%) | 3 (18.8%) | 0.908 |
| Rebleed | 19 (12.9%) | 3 (11.1%) | 8 (11.4%) | 4 (26.7%) | 1 (6.3%) | 0.328 |
| Meningitis | 10 (6.8%) | 4 (14.8%) | 4 (5.7%) | 1 (6.7%) | 1 (6.3%) | 0.503 |
| Pneumonia | 9 (6.1%) | 2 (7.4%) | 4 (5.7%) | - | 2 (12.5%) | 0.539 |
| **Supplementary Table 2.** This table shows baseline clinical and demographic characteristics stratified by aneurysm treatment modality: surgical clipping, coiling, stent-assisted coiling and flow diversion. Values are presented as absolute numbers (percentages) or mean (±standard deviation). p-values were calculated using analysis of variance (ANOVA) or the Chi-squared test, as appropriate. Asterisks (*) indicate statistical significance at p < 0.05.  Abbreviations: **WFNS** World Federation of Neurological Societies**, EVD E**xternal Ventricular Drain**, ELD** External Lumbar Drain**, VPD** Ventriculoperitoneal Drain, **ACOM** Anterior Communication Artery**, PCOM** Posterior Communicating Artery**, ACA** Anterior Cerebral Artery**, PCA** Posterior Cerebral Artery**, MCA** Middle Cerebral Artery**, ICA** Internal Carotid Artery**, BA** Basilar Artery**, OA** Ophthalmic Artery**, SCA** Superior Cerebellar Artery **, AICA** Anterior Inferior Cerebellar Artery**, PICA** Posterior Inferior Cerebellar Artery**, PA** Pericallosal Artery**, aSAH** Aneurysmal Subarachnoid Haemorrhage**, MI** Myocardial Infarction, **DCI** Delayed Cerebral Ischemia | | | | | | |
