# Supplementary material for: Resource use and in-hospital costs after aneurysmal subarachnoid hemorrhage in the Netherlands
Source: Brain Spine. 2025 Aug 28;5:104400. doi: 10.1016/j.bas.2025.104400 (PMC12451355; doi:10.1016/j.bas.2025.104400)
Supplement: Multimedia component 3 [file mmc3.docx]

| **Supplementary Table 3. Subgroup Analysis of In-hospital Costs and Length of Stay** | | | | | |
| --- | --- | --- | --- | --- | --- |
| **Subgroup** | ***N*** | **Total LOS** | **LOS ICU** | **Total in-hospital Cost** | ***p*-Value** |
| **Age** |  |  |  |  | 0.026* |
| 18-39 | 5 (3.4%) | 13 (13-15) | 2 (0-3) | 25,244; 27,044 (19,800-29,789) |  |
| 40-59 | 61 (41.5%) | 15 (12-22) | 4 (2-7) | 44,915; 34,450 (26,700-54,445) |  |
| 60-79 | 70 (47.6%) | 16 (12-25) | 3 (2-6) | 43,258; 33,701 (23,624-61,138) |  |
| ≥80 | 11 (7.5%) | 8 (3-23) | 2 (0-2) | 23,944; 17,416 (8,247-43,380) |  |
| **Sex** |  |  |  |  | 0.454 |
| Male | 40 (27.2%) | 16 (12-23) | 3 (2-7) | 47,552; 34,865 (24,414-64,402) |  |
| Female | 107 (72.8%) | 15 (10-22) | 3 (2-5) | 39,770; 30,245 (24,167-49,984) |  |
| **Year of Admission** |  |  |  |  | 0.195 |
| 2021 | 22 (15.0%) | 19 (14-32) | 4 (3-7) | 50,040; 42,641 (27,782-63,057) |  |
| 2022 | 47 (32.0%) | 14 (9-18) | 3 (1-5) | 36,418; 27,322 (22,591-46,486) |  |
| 2023 | 64 (43.5%) | 16 (10-21) | 3 (2-5) | 38,816; 32,166 (23,559-49,104) |  |
| 2024 | 14 (9.5%) | 16 (14-45) | 3 (1-9) | 61,481; 38,258 (22,032-87,832) |  |
| **WFNS** |  |  |  |  | 0.041* |
| I | 77 (52.4%) | 15 (13-19) | 3 (1-4) | 34,563; 28,515 (24,131-42,134) |  |
| II | 28 (19.0%) | 17 (13-23) | 3 (1-6) | 45,280; 35,676 (26,645-46,224) |  |
| III | 7 (4.8%) | 29 (15-39) | 10 (6-13) | 65,480; 71,699 (49,421-82,432) |  |
| IV | 9 (6.1%) | 34 (3-46) | 3 (2-6) | 46,033; 49,984 (13,840-70,557) |  |
| V | 26 (17.7%) | 12 (3-25) | 6 (3-11) | 52,139; 44,344 (19,635-72,354) |  |
| **Hunt & Hess** |  |  |  |  | 0.058 |
| I | 46 (31.3%) | 15 (13-17) | 3 (0-4) | 31,811; 27,175 (22,231-36,195) |  |
| II | 47 (32.0%) | 16 (12-20) | 3 (1-4) | 43,393; 34,735 (26,910-46,486) |  |
| III | 16 (10.9%) | 17 (11-37) | 3 (2-10) | 50,415; 45,588 (27,613-69,200) |  |
| IV | 8 (5.4%) | 27 (6-35) | 5 (2-6) | 45,273; 45,920 (27,197-59,673) |  |
| V | 30 (20.4%) | 12 (3-27) | 5 (3-11) | 49,530; 41,957 (18,161-71,034) |  |
| **Modified Fisher** |  |  |  |  | 0.003* |
| 0 | 5 (3.4%) | 9 (6-19) | 2 (0-4) | 32,988; 26,445 (16,855-52,393) |  |
| 1 | 39 (26.5%) | 14 (12-16) | 2 (0-3) | 29,004; 27,255 (21,136-33,281) |  |
| 2 | 20 (13.6%) | 16 (12-20) | 3 (0-4) | 36,711; 34,241 (25,509-44,522) |  |
| 3 | 31 (21.1%) | 17 (13-26) | 4 (3-8) | 47,970; 43,981 (29,631-68,001) |  |
| 4 | 52 (35.4%) | 16 (4-29) | 4 (2-9) | 50,771; 40,809 (26,561-71,010) |  |
| **Parenchymal Haemorrhage** |  |  |  |  | 0.343 |
| No | 127 (86.4%) | 15 (12-21) | 3 (2-4) | 40,830; 30,489 (24,901-48,153) |  |
| Yes | 20 (13.6%) | 16 (6-34) | 5 (3-11) | 48,605; 35,676 (24,077-71,423) |  |
| **Subdural Haemorrhage** |  |  |  |  | 0.016* |
| No | 143 (97.3%) | 15 (12-22) | 3 (2-5) | 41,113; 30,489 (24,095-48,849) |  |
| Yes | 4 (2.7%) | 31 (16-48) | 8 (5-11) | 69,574; 68,122 (54,187-86,415) |  |
| **Location Aneurysm** |  |  |  |  | 0.960 |
| Anterior Circulation | 121 (82.3%) | 15 (12-22) | 3 (2-6) | 41,125; 30,723 (24,131-50,989) |  |
| Posterior Circulation | 26 (17.7%) | 15 (7-23) | 2 (0-5) | 45,438; 33,514 (24,265-50,907) |  |
| **Size of the Aneurysm** |  |  |  |  | 0.278 |
| Small (<5mm) | 59 (40.1%) | 15 (12-22) | 3 (2-6) | 40,585; 34,735 (24,901-50,367) |  |
| Medium (5mm-10mm) | 64 (43.5%) | 15 (9-25) | 3 (2-6) | 38,417; 28,685 (22,286-47,737) |  |
| Large (>10mm) | 24 (16.3%) | 16 (8-22) | 3 (2-5) | 54,345; 37,379 (27,889-68,121) |  |
| **Type of Aneurysm** |  |  |  |  | 0.916 |
| Saccular | 122 (83.0%) | 15 (12-24) | 3 (2-6) | 42,771; 30,972 (24,083-52,314) |  |
| Dissection | 17 (11.6%) | 16 (12-18) | 2 (0-6) | 38,212; 34,450 (24,238-46,374) |  |
| Other | 8 (5.4%) | 14 (12-17) | 1 (0-3) | 36,233; 27,885 (26,589-57,461) |  |
| **Smoking status** |  |  |  |  | 0.977 |
| No | 71 (48.3%) | 16 (10-26) | 3 (2-6) | 44,841; 35,489 (22,591-61,408) |  |
| Yes | 53 (36.1%) | 15 (13-21) | 3 (2-5) | 41,093; 30,278 (25,208-48,256) |  |
| Former | 15 (10.2%) | 16 (9-23) | 3 (2-4) | 38,719; 34,735 (25,256-48,849) |  |
| **In-hospital Mortality** |  |  |  |  | 0.021* |
| No | 115 (78.2%) | 16 (14-26) | 3 (2-5) | 43,907; 34,450 (26,556-49,984) |  |
| Yes | 32 (21.8%) | 4 (3-13) | 3 (2-6) | 34,630; 24,474 (14,256-61,732) |  |
| **History of aSAH** |  |  |  |  | 0.059 |
| No | 138 (93.9%) | 15 (12-22) | 3 (2-5) | 41,280; 30,261 (24,083-48,992) |  |
| Yes | 9 (6.1%) | 17 (13-26) | 3 (1-6) | 51,200; 50,367 (38,245-65,720) |  |
| **History of Hypertension** |  |  |  |  | 0.082 |
| No | 97 (66.0%) | 15 (12-22) | 3 (2-5) | 38,748; 29,264 (22,587-47,128) |  |
| Yes | 50 (34.0%) | 17 (12-28) | 3 (2-6) | 47,979; 39,560 (26,364-62,166) |  |
| **DCI** |  |  |  |  | <0.001* |
| No | 115 (78.2%) | 15 (9-18) | 3 (1-4) | 33,927; 27,833 (22,271-41,754) |  |
| Yes | 32 (21.8%) | 24 (16-46) | 8 (3-11) | 70,494; 65,583 (44,921-84,447) |  |
| **Rebleed** |  |  |  |  | 0.755 |
| No | 128 (87.1%) | 16 (12-22) | 3 (2-5) | 39,981; 31,195 (24,990-48,675) |  |
| Yes | 19 (12.9%) | 14 (4-31) | 4 (1-12) | 54,735; 33,281 (21,567-71,699) |  |
| **Hydrocephalus** |  |  |  |  | <0.001* |
| No | 85 (57.8%) | 15 (12-17) | 3 (1-4) | 31,719; 27,292 (21,962-38,651) |  |
| Yes | 62 (42.2%) | 21 (12-32) | 4 (3-9) | 55,829; 45,180 (28,345-72,241) |  |
| **Meningitis** |  |  |  |  | <0.001* |
| No | 137 (93.2%) | 15 (11-21) | 3 (2-5) | 38,883; 30,245 (23,714-47,962) |  |
| Yes | 10 (6.8%) | 34 (18-60) | 9 (3-16) | 83,058; 72,276 (40,076-100,582) |  |
| **Pneumonia** |  |  |  |  | 0.001* |
| No | 138 (93.9%) | 15 (11-21) | 3 (2-5) | 40,441; 30,192 (23,881-47,866) |  |
| Yes | 9 (6.1%) | 25 (17-38) | 9 (3-13) | 64,066; 60,321 (46,876-76,515) |  |
|  | | | | | |

**Legend**

**Supplementary Table 3.** This table provides a subgroup analysis of total length of stay, intensive care unit length of stay and total in-hospital costs across various demographic, clinical and radiological parameters. This table is an extended version of Table 4. Values are reported as: absolute numbers (percentages), median (IQR 25-75) or mean; median (IQR 25-75). *p*-values were calculated using the Mann-Whitney U test and the Kruskal-Wallis test to compare the total in-hospital costs. Asterisks (*) indicate statistical significance at p < 0.05. Costs are reported in 2024 Euros and rounded to the nearest Euro.

Abbreviations: **WFNS** World Federation of Neurosurgical Societies, **aSAH** Aneurysmal Subarachnoid Haemorrhage, **DCI** Delayed Cerebral Ischemia
